# Low P-Selectin Glycoprotein Ligand-1 Expression in Neutrophils Associates with Disease Activity and Deregulated NET Formation in Systemic Lupus Erythematosus

**DOI:** 10.3390/ijms24076144

**Published:** 2023-03-24

**Authors:** Antonio Muñoz-Callejas, Elena González-Sánchez, Javier Silván, Esther San Antonio, Rafael González-Tajuelo, Alejandra Ramos-Manzano, Inés Sánchez-Abad, Isidoro González-Alvaro, Javier García-Pérez, Eva G. Tomero, Rosario García de Vicuña, Esther F. Vicente-Rabaneda, Santos Castañeda, Ana Urzainqui

**Affiliations:** 1 Immunology Department, Fundación de Investigación Biomédica (FIB), Instituto de Investigación Sanitaria-Princesa (IIS-Princesa), Hospital de la Princesa, 28006 Madrid, Spain; 2 Rheumatology Department, Fundación de Investigación Biomédica (FIB), Instituto de Investigación Sanitaria-Princesa (IIS-Princesa), Hospital de la Princesa, 28006 Madrid, Spain; 3 Pulmonology Department, Fundación de Investigación Biomédica (FIB), Instituto de Investigación Sanitaria-Princesa (IIS-Princesa), Hospital de la Princesa, 28006 Madrid, Spain; 4 Catedra UAM-Roche, EPID-Future, Department of Medicine, Universidad Autónoma de Madrid, 28049 Madrid, Spain

**Keywords:** PSGL-1, P-selectin, neutrophils, SLE pathogenesis, NETs

## Abstract

Systemic Lupus Erythematosus (SLE) is an autoimmune disease characterized by the generation of anti-DNA autoantibodies due to exposure of immune cells to excessive amounts of extracellular DNA. Lack of P-selectin in mice induces the development of a lupus-like syndrome and patients with cutaneous lupus have reduced P-selectin expression in skin vessels. Using flow cytometry we analyzed in healthy donors and patients the expression of P-selectin Glycoprotein Ligand-1 (PSGL-1) in circulating neutrophils and the implication of PSGL-1/P-selectin interaction in neutrophil extracellular traps (NETs) generation. We found a statistical significance that neutrophils from active SLE patients have a reduced expression of PSGL-1 and low levels of PSGL-1 in neutrophils from SLE patients associated with the presence of anti-dsDNA antibodies, clinical lung involvement, Raynaud’s phenomenon, and positive lupus anticoagulant. PSGL-1 is present along the DNA in the NET. In healthy donors, neutrophil interaction with immobilized P-selectin triggers Syk activation, increases the NETs percentage and reduces the amount of DNA extruded in the NETs. In active SLE patients, neutrophil interaction with P-selectin does not activate Syk or reduce the amount of DNA extruded in the NETs, that might contribute to increase the extracellular level of DNA and hence, to disease pathogenesis.

## 1. Introduction

Neutrophils are the first line of defense against pathogens. They are professional phagocytic cells and release bactericidal molecules, cytokines, and chemokines [1]. They can also extrude their nuclear DNA to the extracellular medium, forming networks of DNA decorated with nuclear and cytoplasmic proteins, known as NETs (neutrophil extracellular traps) [2].

Systemic lupus erythematosus (SLE) is a severe systemic connective tissue autoimmune disease that can affect any organ and has phases of remission/inactivity and peaks or outbreaks of activity. Its highest prevalence occurs in women of reproductive age [3] and has no curative treatment. SLE is characterized by the loss of regulation of the innate and adaptive immune response, and the lack of tolerance to self-antigens leading to the generation of autoantibodies against cytoplasmic and nuclear antigens, including DNA [4]. It has been proposed that the generation of anti-DNA antibodies can be the consequence of a defective elimination of NETs [5,6,7]. Importantly, SLE patients with renal involvement have higher levels of NETs in serum, and lower DNase I activity, suggesting that a greater presence of NETs is a factor involved in the pathogenesis of this autoimmune disease [5,8].

P-selectin is a transmembrane protein, present in platelet and human endothelial cells [9]. PSGL-1, a mucin-like transmembrane glycoprotein present in all types of leukocytes, is the main ligand for P-selectin and also binds to the other selectins (E- and L-selectin), although with lower affinity [10,11]. The interactions of PSGL-1 with P- and E- selectins are responsible for the tethering and rolling of leukocytes on endothelial cells during the first steps of extravasation. These interactions are also responsible for the activation of integrins for leukocyte firm adhesion before tissue extravasation [12]. Furthermore, our group described that during these interactions, PSGL-1 triggers a signaling cascade initiated by Syk [13], a tyrosine kinase involved in triggering the generation of NETs [14,15,16]. In monocyte-derived-dendritic cells, PSGL-1 signaling cascade promotes the capacity of these cells to induce the differentiation of naïve T cells to T-regulatory (Treg) cells [17]. This process helps to maintain the immune system homeostasis in environments such as the colonic lamina propria in mice [18]. In accordance with these data, the absence of PSGL-1 in mice induces an autoimmune syndrome similar to human scleroderma [19] and the absence of P-selectin induces an autoimmune syndrome similar to human SLE [20].

Remarkably, SLE patients with lupus nephritis have high levels of P-selectin in urine, which correlate with disease severity [21], and patients with cutaneous lupus have low levels of P-selectin in the endothelium of skin blood vessels [20]. Moreover, it has been described in mice that platelet P-selectin promotes the formation of NETs in a PSGL-1-dependent way [22]. Regarding human neutrophils, there is no clear evidence regarding the role of P-selectin in the NET generation [23,24].

Understanding the pathogenesis of SLE is crucial to find new molecular targets for new treatment strategies. With the aim of contributing at elucidating the mechanisms implicated in SLE pathogenesis, in the present study we studied the involvement of PSGL-1/P-selectin interaction in controlling the formation of NETs. We also studied the alterations present in SLE patients, since they could potentially contribute to the pathogenesis of SLE.

## 2. Results

### 2.1. PSGL-1 Expression Is Reduced in Neutrophils of Active SLE Patients

**Figure 1:**
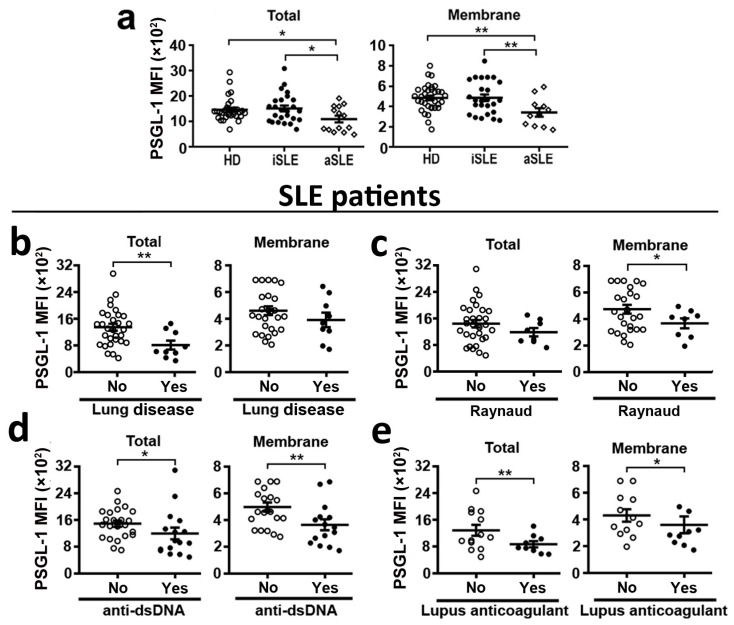
PSGL-1 expression in circulating neutrophils from SLE patients and healthy donors. Expression of PSGL-1 was analyzed by flow cytometry. (**a**) Total (left panel) and membrane (right panel) expression of PSGL-1 in neutrophils from healthy donors (HD, *n* = 41), inactive SLE patients (iSLE, *n* = 33), and active SLE patients (aSLE, *n* = 14). (**b**–**e**) Association of membrane and total PSGL-1 expression in neutrophils from SLE patients with the presence of different clinical characteristics: (**b**) presence of anti-dsDNA autoantibodies in serum (*n* = 47), (**c**) lupus anticoagulant positivity (*n* = 25), (**d**) lung disease involvement (*n* = 47), and (**e**) presence of Raynaud´s phenomenon (*n* = 47). Data represent mean fluorescence intensity (MFI) ± SEM. Differences were analyzed by Student’s *t*-test. * *p* ≤ 0.05; ** *p* ≤ 0.01. HD: healthy donors. SLE: Systemic Lupus Erythematosus. iSLE: inactive SLE. aSLE: active SLE.

Our analysis of PSGL-1 expression in neutrophils showed no difference in PSGL-1 expression between patients and healthy donors (HD). However, analyzing separately aSLE and iSLE, we found that neutrophils from aSLE patients had reduced levels of PSGL-1 compared to those from HD (Figure 1a). Interestingly, membrane and total expression levels of PSGL-1 were lower in aSLE patients than in iSLE patients (Figure 1a).

The statistical study of all the SLE patient cohort (iSLE and aSLE patients) showed that low expression levels of PSGL-1 in neutrophils, both total and membrane expression, were associated with the presence of anti-dsDNA autoantibodies (Figure 1b) and positive lupus anticoagulant (LA) (Figure 1c). In addition, low PSGL-1 expression levels in neutrophils were associated with the presence of clinical lung involvement (Figure 1d), and the presence of Raynaud´s phenomenon (Figure 1e).

### 2.2. PSGL-1 Is Located in the NETs

We checked the presence of PSGL-1 in the NETs generated by neutrophils that were obtained from HD given that: PSGL-1 is a receptor of several pathogens [25,26,27,28,29,30,31,32], the relevance of NETs in pathogen elimination, and their possible implication in SLE pathogenesis.

**Figure 2:**
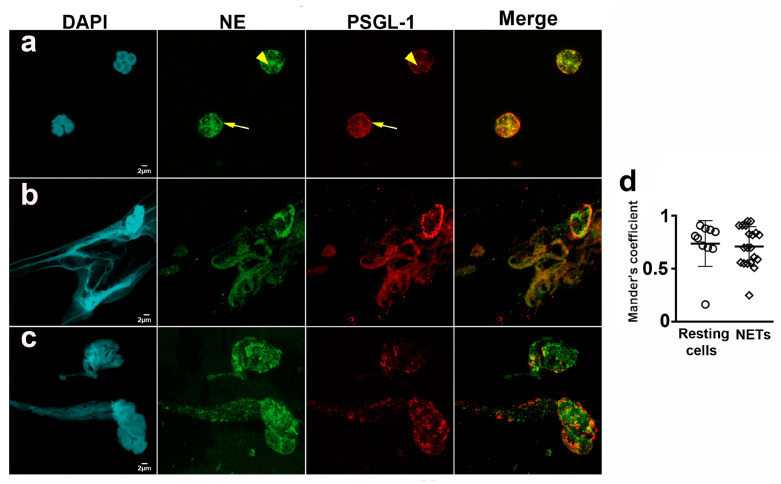
PSGL-1 localization in neutrophils and NETs. Representative microphotographs of neutrophils isolated from blood of HD, after 1 h incubation on BSA and labeled with anti-PSGL-1 monoclonal antibody plus anti-mouse-Alexa Fluor555 (red) secondary antibody, anti-neutrophil elastase rabbit polyclonal antibody plus anti-rabbit-Alexa Fluor 647 (green) secondary antibody and DAPI to visualize DNA (cyan). (**a**) Resting neutrophils. (**b**,**c**) Spontaneous NETs. (**d**) Scatterplot representing Mander’s coefficient for PSGL-1 and NE co-localization in resting neutrophils (0.74 ± 0.21) and NETs (0.67 ± 0.21). Scale bar = 2 µm. All images were obtained by confocal microscopy with 63× oil objective and a 5× digital zoom. Arrowheads indicate cytoplasmic granules. Arrows indicate the neutrophil membrane. NETs: Neutrophil extracellular traps. NE: Neutrophil elastase. PSGL-1: P-selectin glycoprotein ligand-1.

Neutrophils were labeled with DAPI, anti-neutrophil elastase and anti-PSGL-1 antibodies. Spontaneous NETs, neutrophil elastase and PSGL-1 were visualized by confocal microscopy. We analyzed the presence of PSGL-1 in resting neutrophils (Figure 2a) and in NETs (Figure 2b,c), and found that PSGL-1 was localized on the membrane and in cytoplasmic granules of resting cells (Figure 2a). Interestingly, PSGL-1 partially co-localized with NE in the cytoplasmic granules (Mander’s coefficient = 0.74 ± 0.21) (Figure 2a,d). In the NETs, PSGL-1 was localized along the DNA (Figure 2b,c), also partially co-localizing with the NE (Mander’s coefficient 0.67 ± 0.21) (Figure 2b–d).

### 2.3. Neutrophils from SLE Patients Are More Susceptible to Generate NETs

Quantification of extracellular DNA after PMA treatment of HD neutrophils gave similar results with confocal microscopy, fluorometry and flow cytometry. Confocal microscopy showed increased percentage of NETosing cells after PMA treatment (Supplementary Figure S1a,b). The increment in the percenteage of NETs was also obtained by flow cytometry analysis of Sytox Green/NE double positive events (Supplementary Figure S1c). Fluorescence at 485 nm showed an increment of extracellular DNA intensity after PMA activation (Supplementary Figure S1d). The same tendency was obtained by flow cytometry as Sytox Green mean fluorescence intensity (MFI) fold induction of Sytox Green/NE double positive events (Supplementary Figure S1e).

**Figure 3:**
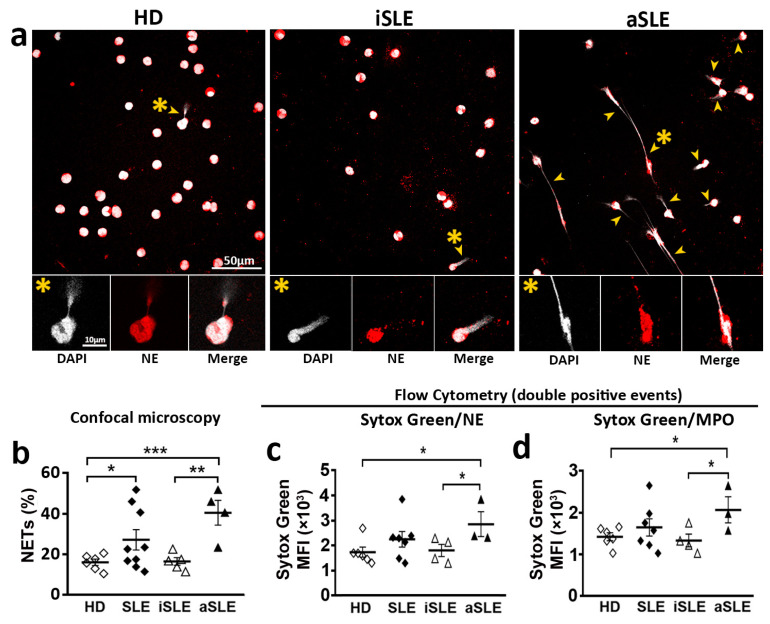
Susceptibility to NET generation of neutrophils from SLE patients and healthy donors in basal experimental conditions. Quantification of NETs generated by neutrophils from HD, iSLE patients and aSLE patients. (**a**) Representative fields of DAPI/NE stained unstimulated neutrophils from HD, iSLE and aSLE obtained by confocal microscopy with a 63x objective. Scale bar = 10–50 µm. Yellow arrowheads indicate NETs. Yellow asterisks indicate NETosing cells amplified below the image. (**b**) Percentage of NETs quantified by confocal microscopy. (**c**,**d**) MFI of Sytox Green in (**c**) Sytox Green/NE-APC and (**d**) Sytox Green/MPO-PE double positive events, respectively. Statistical analysis was performed using unpaired Student’s *t*-test and Mann-Whitney test. * *p* ≤ 0.05; ** *p* ≤ 0.01, *** *p* ≤ 0.001. Data show mean fluorescence intensity (MFI) ± SEM or percentage ± SEM. HD (*n* = 6–12), iSLE (*n* = 4–7), aSLE (*n* = 3–8). HD: healthy donors. SLE: Systemic Lupus Erythematosus. iSLE: inactive SLE. aSLE: active SLE. NETs: Neutrophil extracellular traps. NE: Neutrophil Elastase. MPO: Myeloperoxidase. SLE = Total population of patients included.

The percentage of NETs generated by neutrophils was higher in SLE patients than in HD (Figure 3a,b). A more detailed study showed that aSLE generated higher percentage of NETs compared to HD and iSLE patients (Figure 3a,b). Moreover, the MFI of NETs generated by neutrophils from aSLE patients was higher than that of NETs from HD and iSLE patients (Figure 3c,d), indicating that the amount of extracellular DNA extruded by aSLE cells is higher than that extruded by neutrophils from HD and iSLE patients (Figure 3c,d). No differences were found between HD and iSLE patients.

### 2.4. PSGL-1/P-Selectin Interaction Modulates NET Generation in HD

**Figure 4:**
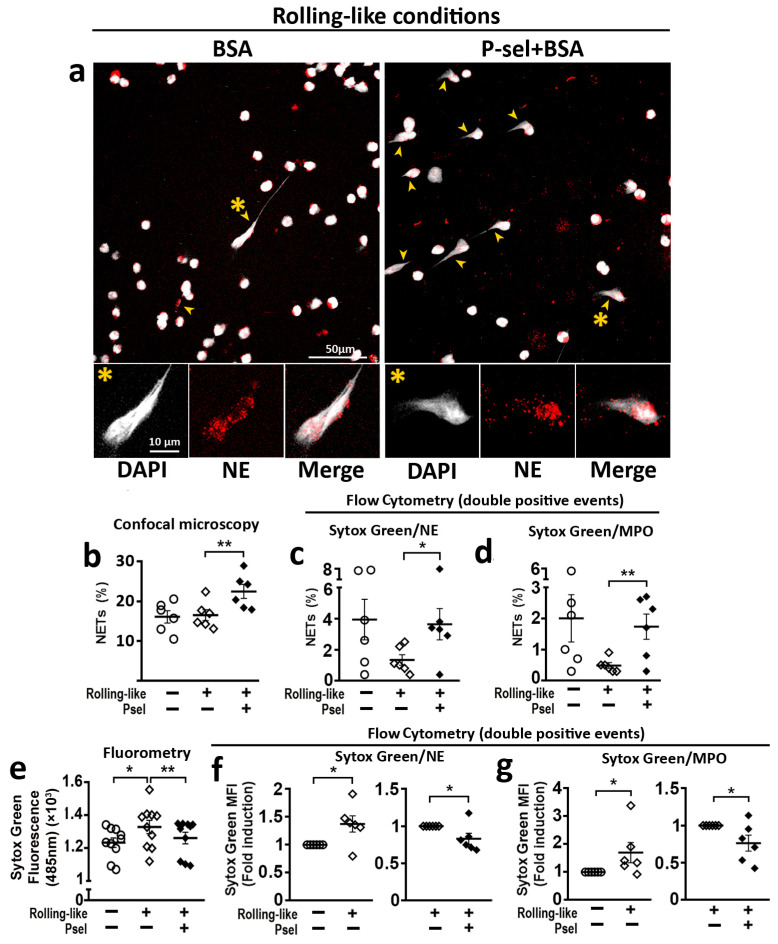
Interaction of HD neutrophils with P-selectin regulates NET generation. Peripheral neutrophils isolated from healthy donors were incubated in rolling-like conditions. (**a**) Representative confocal microphotographs taken with 63× and 63× plus 5× digital zoom of DAPI/NE staining neutrophils in rolling-like experimental conditions on BSA or BSA+P-selectin. Scale bar = 10–50 µm. Yellow arrowheads indicate NETs. Yellow asterisks indicate NETosing cells amplified below the image. (**b**) Percentage of NETs quantified by confocal microscopy. (**c**,**d**) Percentage of NETosing cells in (**c**) Sytox Green/NE-APC and (**d**) Sytox Green/MPO-PE double positive events, respectively. (**e**) Extracellular Sytox Green MFI analyzed by fluorometry at 485 nm. (**f**,**g**) Sytox Green MFI fold induction in (**f**) Sytox Green/NE-APC and (**g**) Sytox Green/MPO-PE double positive events, respectively. Data are presented as MFI fold induction or percentage ± SEM. Statistical analysis was performed using paired Student’s *t*-test and Wilcoxon test. * *p* ≤ 0.05; ** *p* ≤ 0.01; *n* = 6–12. NETs: Neutrophil extracellular traps. HD: healthy donors. NE: Neutrophil elastase. MPO: Myeloperoxidase.

To analyze the effect of PSGL-1/P-selectin interaction on the generation of NETs, circulating neutrophils were incubated in rolling-like conditions in the presence or absence of P-selectin. Confocal microscopy (Figure 4a,b) and flow cytometry (Figure 4c,d) showed that P-selectin increased the percentage of NETs generated by neutrophils. Fluorometry assays showed that rolling on BSA before adhesion increased the amount of extracellular DNA while the presence of P-selectin impaired this increase (Figure 4e). Similar reduction of extracellular DNA (Sytox Green MFI) by P-selectin was observed by flow cytometry analysis with Sytox Green/Neutrophil Elastase (Figure 4f) or Sytox Green/Myeloperoxidase (Figure 4g) double labeling of non-permeabilized cells.

### 2.5. PSGL-1/P-Selectin Interaction Does Not Control DNA Extrusion in SLE Patients with Active Disease

**Figure 5:**
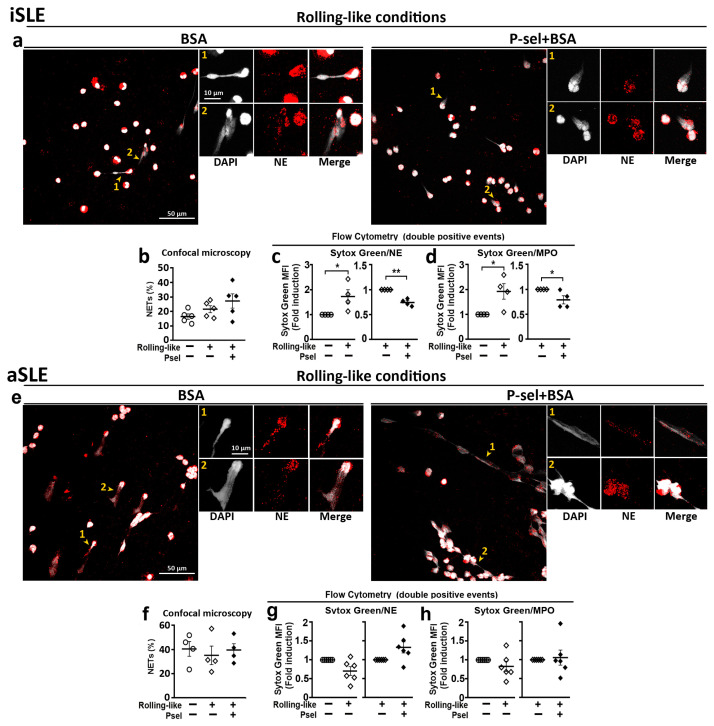
Deregulated NET generation by neutrophils from SLE patients. Neutrophils from inactive (iSLE) and active (aSLE) patients were incubated in rolling-like conditions in the presence of BSA or BSA+P-selectin and NETs were quantified by confocal microscopy and by flow cytometry. (**a**–**d**) iSLE patients: (**a**) Representative confocal microphotographs of DAPI/NE stained neutrophils. (**b**) Quantification of NETs by confocal microscopy. (**c**,**d**) MFI fold induction of Sytox Green in (**c**) Sytox Green/NE double positive events and (**d**) Sytox Green/MPO double positive events. (**e**–**h**) aSLE patients: (**e**) Representative confocal microphotographs of DAPI/NE stained neutrophils. **(f**) Quantification of NETs by confocal microscopy. (**g**,**h**) MFI fold induction of Sytox Green in (**g**) Sytox Green/NE double positive events and (**h**) Sytox Green/MPO double positive events. Confocal images were taken with 63× and 63× plus 5× digital zoom. Scale bar = 10–50 µm. Yellow arrowheads indicate NETs. Yellow numbers 1 and 2 indicate NETosing cells amplified next to the 63× image. Data are presented as mean fluorescence intensity (MFI) fold induction or percentage ± SEM. Statistical analysis was performed using paired Student’s *t*-test and Wilcoxon test. * *p* ≤ 0.05, ** *p* ≤ 0.01. iSLE patients (*n* = 4–7), aSLE patients (*n* = 6–8). SLE: Systemic Lupus Erythematosus. iSLE: inactive SLE. aSLE: active SLE. NETs: Neutrophil extracellular traps.

The analysis of NET generation by neutrophils obtained from SLE patients showed that in the case of iSLE patients, the interaction with P-selectin did not increase the percentage of NETs generated by neutrophils (Figure 5a,b) although it reduced the amount of expelled extracellular DNA (Figure 5c,d). In the case of aSLE neutrophils, P-selectin interaction did not increase the percentage of NETs (Figure 5e,f) and did not reduce the amount of extruded DNA (Figure 5g,h).

### 2.6. PSGL-1/P-Selectin Interaction Signaling Is Altered in SLE Patients

**Figure 6:**
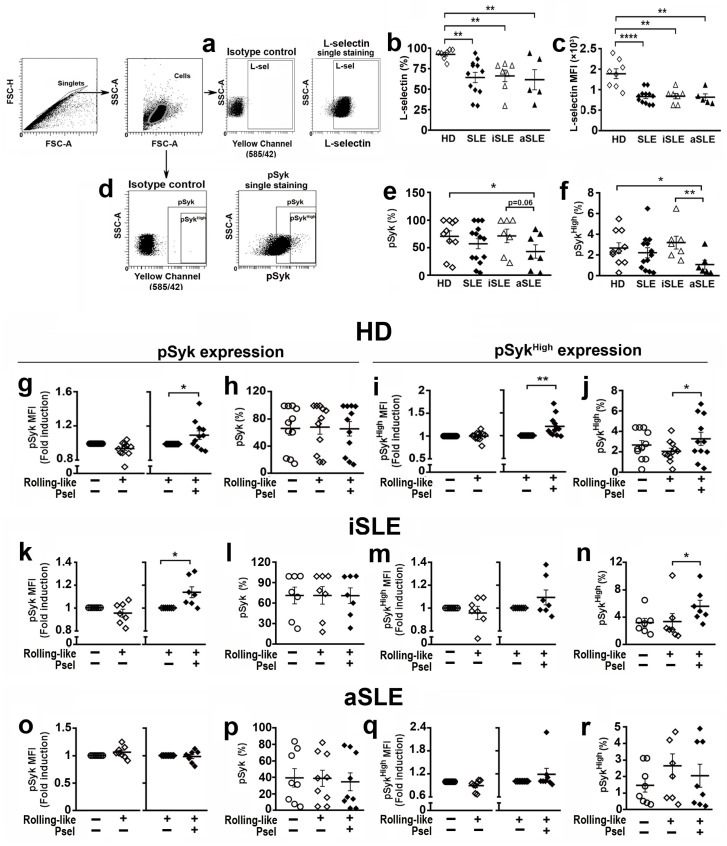
Expression of L-selectin and pSyk in neutrophils from healthy donors and SLE patients in experimental conditions. (**a**,**d**) Gating strategy for identification of (**a**) L-selectin, and (**d**) pSyk and pSyk^High^ populations: Singlets were identified by FSC-H and FSC-A criteria. Neutrophil populations were identified in the singlets gate by SSC-A/FSC-A criteria. Gates for positive cells were identified by using isotype controls. (**b**,**c**) L-selectin expression in basal experimental conditions: (**b**) Percentage of cells expressing L-selectin, and (**c**) MFI of L-selectin. (**e**,**f**) Percentage of neutrophils expressing (**e**) pSyk or (**f**) pSyk^High^ in basal experimental conditions. (**g**–**j**) pSyk and pSyk^High^ expression in HD neutrophils in rolling-like conditions: (**g**) MFI fold induction of pSyk, (**h**) Percentage of pSyk expressing neutrophils, (**i**) MFI of pSyk^High^, and (**j**) Percentage of pSyk^High^ expressing neutrophils. (**k**–**n**) pSyk and pSyk^High^ expression in iSLE neutrophils in rolling-like conditions: (**k**) MFI fold induction of pSyk, (**l**) Percentage of pSyk expressing neutrophils, (**m**) MFI fold induction of pSyk^High^, and (**n**) Percentage of pSyk^High^ expressing neutrophils. (**o**–**r**) pSyk and pSyk^High^ expression in aSLE neutrophils in rolling-like conditions: (**o**) MFI fold induction of pSyk, (**p**) Percentage of pSyk expressing cells, (**q**) MFI fold induction of pSyk^High^, and (**r**) Percentage of pSyk^High^ expressing neutrophils. Data are presented as MFI fold induction or percentage ± SEM. Statistical analysis was performed using paired Student’s *t*-test and Wilcoxon test. * *p* ≤ 0.05; ** *p* ≤ 0.01, **** *p* ≤ 0.0001. HD (*n* = 7–12). iSLE patients (*n* = 7), aSLE patients (*n* = 7–8). MFI: Mean fluorescence intensity. HD: Healthy donor. SLE: Systemic Lupus Erythematosus. iSLE: inactive SLE. aSLE: active SLE. SLE: Total population of patients included. Open circles: basal conditions. Open diamonds: rolling-like on BSA. Black diamonds: rollink-like on P-selectin.

Syk has been implicated in NET generation [14,15,16]. Given that PSGL-1 interaction with P-selectin induces Syk activation [13] and L-Selectin contributes to full activation of neutrophils, when interacting with P-selectin [33], we first studied the expression level of L-Selectin in the cell membrane and the level of pSyk in basal experimental conditions of neutrophils from HD and SLE patients. We found that SLE patients showed reduced percentage of neutrophils expressing L-selectin in the membrane (Figure 6b) as well as lower expression level of L-selectin with respect to HD, regardless of disease activity (Figure 6c). In addition, we found that the percentage of neutrophils with pSyk and pSyk^High^ expression was lower in aSLE patients compared to HD and iSLE patients (Figure 6e,f).

Regarding neutrophils from HD, the interaction with P-selectin in rolling-like conditions increased the phosphorylation level of pSyk (Figure 6g), and pSyk^High^ (Figure 6i), including the percentage of pSyk^High^ neutrophils (Figure 6j). This, however, did not change the percentage of pSyk expressing cells (Figure 6h).

In the case of iSLE patients, the interaction of neutrophils with P-selectin increased pSyk levels (Figure 6k) as well as the percentage of cells with pSyk^High^ (Figure 6n), but not the percentage of pSyk expressing cells (Figure 6l) nor the expression level of pSyk^High^ (Figure 6m). In aSLE patients, neutrophils (Figure 6o–r) did not show any increment of Syk activity in response to P-selectin interaction.

## 3. Discussion

In the present study we found that the expression of PSGL-1 is reduced in neutrophils from SLE patients during disease activity (Figure 1a) and that low PSGL-1 expression in neutrophils associates with the presence of anti-dsDNA antibodies (Figure 1b), which in turn associates with disease activity (Table 1 and Table 2). We have also found that low PSGL-1 expression in neutrophils associates with positive Lupus anticoagulant (LA) (Figure 1c), which leads to increased clotting time. This is a controversy that occurs in patients with a high tendency to form thrombus and for which there is no explanation [34,35]. PSGL-1 is implicated in thrombi formation [36,37] and our data suggest that low PSGL-1 expression could be contributing to LA positivity, coinciding with previous data described in mice [38]. In addition, our data indicate that low PSGL-1 expression in neutrophils is associated with the presence of Raynaud´s phenomenon and clinical lung involvement (Figure 1d,e). Thus suggesting that low PSGL-1 expression could be involved in SLE pathogenesis and pointing PSGL-1 on neutrophils could be used as a diagnostic marker for SLE activity and a potential prognostic marker for lung disease.

Moreover, we also found that PSGL-1 localizes along the DNA in the NETs (Figure 2). Also that the interaction of neutrophils with P-selectin induces Syk activation (Figure 6) and regulates the release of DNA by reducing the amount of DNA extruded in the NET but increasing the percentage of NETs generated (Figure 4). Interestingly, in the case of neutrophils obtained from patients with aSLE, the interaction with P-selectin does not activate Syk and does not reduce the amount of extruded DNA in the NETs (Figure 5 and Figure 6).

DNA extracellular networks have an important role in combating pathogens [39]. When, however, NET generation is out of control, they can have a pathogenic role in diseases such as SLE, by promoting the generation of autoantibodies against DNA and associated proteins [40]. PSGL-1 is a receptor for several pathogens, including: *Streptococcus pneumoniae* [25], *Staphylococcus aureus* [26], *Anaplasma phagocytophilum* [27], *Ehrlichia* sp. [28], enterovirus 71 [29], HIV-1 [30,31] and SARS-CoV-2 [32]. We, therefore, checked for the presence of PSGL-1 in NETs of HD and found it localized along the DNA. This finding suggests that PSGL-1 might contribute to trap and concentrate these pathogens on the network, thus preventing pathogen dissemination and helping to neutralize and eliminate them. Interestingly, PSGL-1 is present in cytoplasmic granules of resting neutrophils, partially co-localizing with neutrophil elastase, a protease implicated in the first steps of NET generation (Figure 2).

In addition, it has been shown that PSGL-1/P-selectin interaction induces NET production in mice [22,37,41], but our work shows for the first time that, in human neutrophils, PSGL-1/P-selectin interaction regulates NET generation. We have found that interaction of human neutrophils with P-selectin increases the percentage of NETs generated by these cells although, importantly, reduces the amount of DNA released in the NETs (Figure 4). This result suggests that PSGL-1/P-selectin axis could be a physiological system to control the DNA release in human neutrophils. Therefore, PSGL-1/P-selectin interaction would contribute to pathogen elimination by increasing the percentage of NET generation, but avoiding excessive DNA accumulation in tissues. Hence limiting the exposure of the immune system to DNA and reducing tissue damage. This result conforms to leukocyte extravasation while eliciting tolerogenic signals to control inflammation. Altogether, these functions support a role of PSGL-1 as a relevant checkpoint for the immune system [10,42].

We also analyzed the susceptibility of neutrophils from lupus patients to generate NETs in our different experiment conditions. Furthermore, we analyzed the level of Syk activation, given that different reports described that the activation of the tyrosine kinase Syk by different stimuli was implicated in the generation of NETs [14,15,16]. In basal conditions, neutrophils from iSLE patients, both the basal susceptibility to NET generation and the amount of DNA extruded in the NETs were similar to HD (Figure 3). Regarding Syk activation in iSLE patients, the basal percentage of pSyk+ and pSyk^High+^ neutrophils was similar to HD (Figure 6e,f). In the case of neutrophils from patients with active disease that express lower levels of PSGL-1, the percentage of neutrophils with pSyk and pSyk^high^ in basal conditions was lower than in HD and less than in iSLE patients (Figure 6e,f). But aSLE neutrophils generated higher percentage of NETs with higher MFI, indicating higher amounts of extracellular DNA extruded by aSLE neutrophils (Figure 3b–d).

In the case of rolling-like conditions, we found that the interaction with P-selectin increases the phosphorylation level of pSyk and pSyk^High^ in neutrophils from HD, but not the percentage of neutrophils with pSyk (Figure 6g–j). Regarding NET generation in HD, the interaction with P-selectin increases the percentage of neutrophils generating NETs but reduces the amount of DNA expelled to the extracellular space. These data suggest that the increase in the phosphorylation level of Syk induced by P-selectin in neutrophils could contribute to reduce the amount of extruded DNA (Figure 4f,g).

Remarkably, our studies in patients with SLE show that P-selectin interaction with neutrophils from iSLE patients, as in the case of HD, reduces the amount of DNA released in NETs, thus controlling DNA extracellular accumulation. However, P-selectin does not increase the percentage of NET generation (Figure 5), which might compromise pathogen elimination in iSLE patients. This would contribute to increased susceptibility to infections observed in these patients [43]. Regarding Syk activation, in the case of neutrophils from iSLE patients, as in HD, the interaction with P-selectin is capable of increasing the expression level of pSyk, which could explain the reduced DNA extruded in the NET. However, despite Syk activation, neutrophils are not fully activated since P-selectin does not increase the percentage of NETs (Figure 6).

Moreover, in the case of neutrophils from aSLE patients, P-selectin does not reduce DNA extrusion, suggesting that the reduction of PSGL-1 expression during disease activity might contribute to DNA accumulation in tissues. In effect contributing to tissue damage and disease pathogenesis, pointing at PSGL-1 as a molecular target during disease activity (Figure 5). Regarding Syk activation, aSLE neutrophil interaction with P-selectin did not induce Syk activation (Figure 6). These data indicate that neutrophils from patients with active disease, in accordance with their lower expression of PSGL-1, are not able to respond to P-selectin engagement, probably because of insufficient PSGL-1 molecules available to trigger Syk activation. Given the association of low PSGL-1 expression with the presence of clinical characteristics such as Raynaud´s phenomenon or lung disease (Figure 1) and the contribution of PSGL-1/P-selectin interaction in the control of NET generation, that we describe in the present paper, both PSGL-1 and P-selectin should be further studied as potential targets for new SLE therapeutic strategies.

Since L-selectin expression is important for full activation of neutrophils by P-selectin [33] and given the lack of response to P-selectin of aSLE neutrophils, we checked the expression of L-selectin in iSLE and aSLE neutrophils in our basal experimental conditions. We found that L-selectin expression was reduced in SLE neutrophils regardless of disease activity (Figure 6). In agreement with a previous report [33], low L-selectin in aSLE neutrophils together with reduced PSGL-1 expression would lead to lack of response to P-selectin. In iSLE neutrophils, although they have a normal level of PSGL-1 expression, the reduced expression of L-selectin would make their full activation difficult.

All these data indicate that the expression level of PSGL-1 in neutrophils and the interaction of this molecule with P-selectin during homeostatic recirculation, for tissue surveillance and cell replenishment, are very important for maintaining neutrophil homeostasis. Understanding cell signaling pathways in autoimmune diseases is challenging and very important to find target molecules for the development of new treatments. Along these lines, Syk has been proposed as a possible therapeutic target for cutaneous lupus [44]. Several Syk inhibitors were also developed to treat autoimmune diseases, such as rheumatoid arthritis, but these have important side effects [45,46]. Our findings also suggest that Syk inhibition during SLE disease activity might be detrimental since aSLE patients show lower percentage of neutrophils with activated Syk and increased NET generation.

In summary, our work in the present study suggests that PSGL-1/P-selectin interaction during the extravasation process to tissue contributes to regulate NET generation. Specifically, PSGL-1/P-selectin interaction promotes NET generation, but reduces the amount of DNA released to the medium in NETs, thus ensuring pathogen elimination without induction of tissue damage. Remarkably, this regulation is lost in SLE patients, especially in aSLE, whose reduced PSGL-1 expression and PSGL-1/P-selectin interaction does not reduce the amount of DNA released in NETs. Paradoxically, these patients have a higher susceptibility to generate NETs.

## 4. Materials and Methods

### 4.1. Subjects

Patients with SLE attending the outpatient Rheumatology clinic at the Hospital Universitario de La Princesa (HUP, Madrid, Spain) were recruited, and then matched with healthy donors (HD) for sex and age. More specifically, two different groups of patients participated in this work. The first group (*n* = 47 SLE patients and 41 HD) participated in the study to analyze the expression of PSGL-1 in neutrophils. The second group (*n* = 15 SLE patients and 12 HD) participated in the in vitro studies to analyze neutrophil functions. Clinical features of patients and their treatment are summarized in Table 1 and Table 2, respectively. The SLE Disease Activity Index (SLEDAI) was used to categorize the activity of the disease: patients with SLEDAI value ≤4 were considered to be in an inactive phase (iSLE), while those with SLEDAI values >4 were in an active phase (aSLE) [47]. Systemic lupus international collaborating clinics/American College of Rheumatology (SLICC/ACR) was used to calculate the cumulative damage index.

All participants signed an informed consent, in accordance with the Declaration of Helsinki. The study was approved by the Ethics Committee for Drug Research of HUP (reference numbers: N° PI758 acta 13/14, N° 3106 and N° 4033). All the experiments and methods were performed in agreement with relevant guidelines and regulations approved by HUP, following the requirements established under Spanish legislation for the field of biomedical research and the protection of personal data.

### 4.2. Labeling of Neutrophils for Flow Cytometry

PSGL-1 expression in blood neutrophils was analyzed by flow cytometry. Blood cells were blocked for 15 min at 4 °C with 10 μg/mL of human γ-globulin (Sigma-Aldrich, St. Louis, MO, USA). Cells were then incubated at 4 °C for 15 min with 1:100 of anti-CD16-APCH7 (BD Biosciences, Rutherford, NJ, USA) and 1:100 anti-PSGL-1-PE (BD Biosciences) antibodies. For intracellular staining, after membrane labeling, cells were incubated for 10 min at room temperature (RT) in FACS lysing solution (BD Biosciences) and then incubated with 1:100 dilution of anti-PSGL-1-PE antibody (BD Biosciences) for 30 min at 4 °C. Finally, cells were washed with PBS+0.5% BSA+5mM EDTA and analyzed on a FACSCanto II (BD Biosciences) cytometer. To determine PSGL-1 positive events, neutrophils were incubated for 15 min at 4 °C with 1:100 IgG-PE isotype control (BD Biosciences) and analyzed in the FACS Canto II cytometer using BD FACSDiva, version 6.1.3 (BD Biosciences, Rutherford, NJ, USA). All the antibodies used in the present study were diluted in PBS and are summarized in Table S1.

L-selectin expression on neutrophils in experimental conditions was analyzed by flow cytometry. Neutrophils, after incubation in basal experimental conditions in Hank’s Balanced Salt Solution (HBSS) (Lonza, Walkersville, MD, USA), were blocked for 15 min at 4 °C with 10 μg/mL of human γ-globulin (Sigma-Aldrich), labeled for 15 min at 4 °C with 1:100 anti-L-selectin-FITC antibody (Biolegend, San Diego, CA, USA) or with 1:100 IgG-FITC isotype control (BD Biosciences) and analyzed on a FACSCanto II cytometer. The percentage of L-selectin expressing cells and expression level of L-selectin, evaluated as Mean Fluorescence Intensity (MFI), were then analyzed. Gating strategy and gate for positivity are shown in Figure 6a.

### 4.3. Neutrophils Isolation

Neutrophils were purified from whole blood from SLE patients and sex and age matched HDs using Histopaque-1077 (PAN-Biotech, Aidenbach, Germany) based density gradient centrifugation at 1800 rpm for 30 min at RT without a break, which separate erythrocytes and granulocytes from mononuclear cells [48]. Then, neutrophils were obtained from the lowest layer and erythrocytes were eliminated by hypotonic lysis with cold water [49,50]. Neutrophil purity was >90%, and confirmed by flow cytometry.

### 4.4. Rolling-like Assays

**Figure 7:**
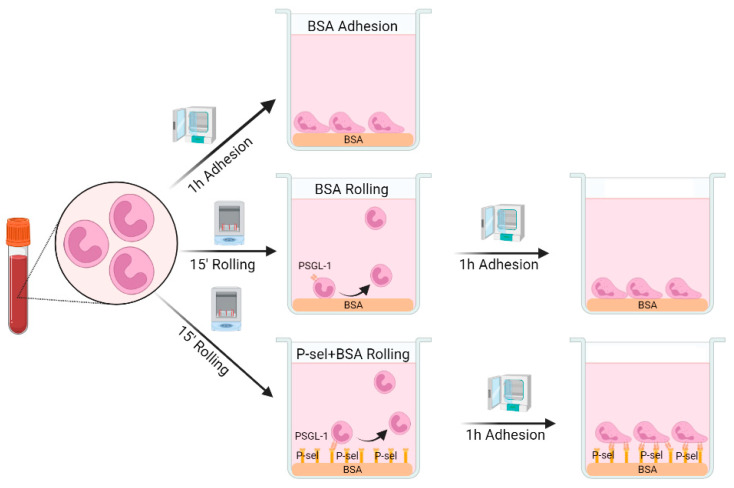
Rolling-like assays in neutrophils isolated from peripheral blood. Created with Biorender.com.

Neutrophils were seeded in HBSS, at a density of 10^6^ cells/mL on plates and coverslips pre-coated with 0.5% BSA for 1 h (Roche Diagnostics, Manheim, Germany) or with 10 µg/mL of recombinant human P-selectin (Bio-Techne, Wiesbaden, Germany) for 1 h and then blocked with 0.5% BSA for 30 min. For the rolling-like conditions, cells were incubated at 37 °C for 15 min at 60 rpm in a shaker, simulating rolling on endothelium, and then incubated for 1 h at 37 °C in a cell incubator, simulating adhesion after rolling (Figure 7) [13,51]. As control, cells were incubated for 1 h at 37 °C on plates/coveslips precoated with 0.5% BSA (basal experimental conditions) (Figure 7).

### 4.5. Activation with PMA

Freshly isolated neutrophils from HD were incubated in HBSS, at a density of 10^6^ cells/mL, for 1 h at 37 °C on plates/coverslips covered with 0.5% BSA in the presence of PMA (Sigma-Aldrich, St. Louis, MO, USA; 25 ng/mL) or without PMA (Non treated cells, NT). Then, cells were processed for NET quantification by confocal microscopy, fluorometry or flow cytometry assays, as described in Section 4.6.

### 4.6. Quantification of NETs by Fluorometry, Confocal Microscopy and Flow Cytometry

To test and validate the performance of flow cytometry for NET quantification in our experimental conditions, fluorometry, flow cytometry and confocal microscopy assays were used to evaluate changes in NET generation induced by PMA activation (Figure S1a–e).

For fluorometry assay, cells were seeded in triplicates (50 × 10^3^ neutrophils/well) at a density of 10^6^ cells/mL and incubated in HBSS in different experimental conditions (rolling-like assays). The cells were then stained with 0.2 μM Sytox Green (Invitrogen, Waltham, MA, USA) for 15 min at 37 °C, washed twice with PBS, and suspended in 50 μL of HBSS. The fluorescence signal was measured at 485 nm emission with a GloMax E7031 Multimicroplate Reader fluorometer (Promega, Madison, WI, USA).

For confocal microscopy assay, neutrophils were incubated on coverslips coated with BSA (0.5%) or 10 μg/mL of P-selectin and then blocked with 0.5% of BSA, in different experimental conditions. Then, cells were fixed with FACS Lysing solution (BD Biosciences) for 5 min at RT, blocked for 30 min at RT with PBS+5% BSA. After blocking, cells were labeled for 1 h at RT with 1:200 dilution of rabbit anti-neutrophil elastase (Abcam, Cambridge, UK) antibody, and then incubated for 30 min at RT with 1:100 dilution of donkey anti-Rabbit IgG Alexa Fluor 647 (Sigma-Aldrich) secondary antibody. Finally, neutrophils were labeled for 5 min at RT with 0.5 μM DAPI (Sigma-Aldrich). Coverslips were then mounted on slides and analyzed by confocal microscopy. Images were acquired by Leica LAS-AF version 2.7.3 software (Leica, Heidelberg, Germany) using 63× oil objective on Leica TCS SP5 confocal microscope (Leica, Heidelberg, Germany) and analyzed with ImageJ/Fiji 1.52e software (Wayne Rasband National Institutes of Health, Bethesda, MD, USA). As described recently [52], we considered NETosing cells when they have decondensed nuclei; they lost the lobular structure of the nucleus; their nuclei have protrussions or they extruded fibers of DNA to the extracellular space labeled with DAPI and NE [2,53] regardless the length, thickness or appearance of the fiber. Neutrophils with clearly lobulated nuclei were considered normal resting neutrophils. Quantification of NETs percentage was performed by counting the number of NETosing cells relative to a total of 100 cells from several microscopy fields obtained with the 63× objective, using ImageJ’s Cell Counter plug-in version 2006/03/06 (University of Sheffield, South Yorkshire, UK).

For flow cytometry analysis, blood isolated neutrophils were suspended in HBSS and incubated under rolling-like experimental conditions (10^6^ cells/mL). Then, cells were removed with 10mM EDTA in PBS, suspended 50 μL de HBSS and labeled for 10 min in the dark at 4 °C with 4 nM Sytox Green. Cells were then washed twice with PBS+0.5% BSA+5mM EDTA. After labeling non-permeabilized cells with Sytox Green, neutrophils were blocked for 15 min at 4 °C with 10 µg/mL human γ-globulin (Sigma-Aldrich) and labeled for 15 min at 4 °C with 1:100 anti-NE-APC monoclonal antibody (Novus Biologicals, Centennial, CO), 1:100 anti-MPO-PE monoclonal antibody (Miltenyi Biotec, Madrid, Spain), 1:100 IgG-APC isotype control (BD Bioscience) or 1:100 IgG-PE isotype control (BD Bioscience). Finally, cells were analyzed by flow cytometry on a BD FACSCanto II using BD FACSDiva, version 6.1.3 (BD Biosciences, Rutherford, NJ, USA). Flow cytometry gives the percentage of Sytox Green/NE or Sytox Green/MPO double positive events, which were considered as NETs. MFI provides reliable information on the expression level of a molecule in a cell population and hence, we considered Sytox Green MFI as an indirect measure of the amount of DNA extruded by neutrophils in each experimental condition. To reduce experimental variability, we normalized the Sytox Green MFI by calculating the ratio of the MFI in each experimental condition relative to the MFI of its corresponding experimental control (MFI fold induction). In the rolling-like condition, MFI fold induction was calculated as a ratio of the MFI of cells incubated in rolling-like conditions on BSA to MFI of cells left to adhere on BSA. MFI NETs induced by P-selectin was calculated as a ratio of MFI of cells incubated on P-sel+BSA in rolling-like conditions relative to MFI of cells incubated on BSA in rolling like conditions.

Gating strategy to identify NETs is summarized in Supplementary Figure S2. Given that cells lose their SSC-A/FSC-A characteristics after DNA release, NETs were analyzed in all forward scatter/side scatter events detected by the cytometer, using Sytox green as DNA dye and NET-specific markers such as neutrophil elastase (NE) [2,53] or myeloperoxidase (MPO) [54,55,56]. NETs were identified as Sytox Green/NE or Sytox Green/MPO double positive events and using IgG-APC and IgG-PE as isotype controls, respectively. For correct compensation, identification of any unspecific binding of flaws in our panels and for proper gating strategy, we used unstained cells (signal given by unstained cells in the Green (530/30)/Red (660/20) or Yellow (585/42) channels), Fluorescence Minus One (FMO) (signal given in the Green channel (530/30) by cells stained with NE and MPO), and single color controls. For single color control, cells were stained with Sytox Green, as described above, and the MFI in the Green channel (530/30) was analyzed in the FACS Canto Cytometer. Information about fluorophores and dyes is shown in Table S1.

### 4.7. Phospho-Syk Measurement by Flow Cytometry

Syk activation was assessed by flow cytometry determination of the expression level of Syk phosphorylated at tyrosines 525 and 526 (pSyk). After incubation in rolling-like conditions, cells were collected, blocked for 15 min at 4 °C with 10 µg/mL human γ-globulin (Sigma-Aldrich), permeabilized 10 min at RT with FACS Lysing solution and labeled for 15 min at 4 °C with 1:200 anti-pSyk-PE antibody (Cell Signaling, Danvers, MA, USA). Cells were then analyzed with a BD FACSCanto II (BD Biosciences) using BD FACSDiva, version 6.1.3 (BD Biosciences). To identify pSyk staining, cells were incubated for 15 min at 4ºC with IgG-PE isotype control (1:100 dilution) and analyzed in the FACS Canto II cytometer. Gating strategy is shown in Figure 6d. Information about the antibodies is shown in Table S1. The percentage of cells expressing high levels of pSyk (pSyk^High^) were also analyzed, including the level of pSyk expression. Changes in the pSyk expression level were assessed by MFI fold induction: (1) fold induction of pSyk MFI positive neutrophils in rolling-like conditions on BSA with respect to adhesion on BSA, and (2) fold induction of pSyk MFI of positive cells in rolling-like conditions on BSA+P-selectin regarding rolling-like conditions on BSA.

### 4.8. Confocal Image Analysis of NETs

For confocal microscopy analysis, after incubation on coverslips coated with BSA, in different experimental conditions, neutrophils were fixed for 5 min at RT with FACS Lysing solution (BD Biosciences) and blocked for 30 min at RT with 5% BSA in PBS. Then, cells were labeled for 1 h at RT with 1:100 dilution of KPL-1 mouse anti-PSGL-1 antibody (1:100, Biolegend) and 1:200 dilution of rabbit anti-neutrophil elastase (Abcam) antibody. Then, cells were incubated for 30 min at RT with 1:100 dilution of Donkey anti-Mouse IgG Alexa Fluor 555 and 1:100 dilution of donkey anti-Rabbit IgG Alexa Fluor 647 (Sigma-Aldrich) secondary antibodies. Finally, cells were stained for 5 min at RT with 0.5 μM DAPI, coverslips were mounted on slides, and then analyzed by confocal microscopy. Images were acquired by Leica LAS-AF 2.7.3 software using 63× oil objective on Leica TCS SP5 confocal microscope (Leica) and analyzed with ImageJ/Fiji 1.52e software. For colocalization analysis, Mander’s overlap coefficients for PSGL-1 and NE staining were analyzed by acquiring the whole stack of 100 cells and 20 NETs, in basal condition, with an inter-plane spacing of each cell or NET on the Z-axis of 0.29 µm, using ImageJ’s JACoP plug-in version 2006/05/31 (Institut Curie, Orsay, France) [57]. Reagents are listed in Table S1.

### 4.9. Statistical Analysis

Data were analyzed using Prism 8.01 (GraphPad Software, La Jolla, CA, USA). First, we assessed the Normality of the samples by using the Shapiro–Wilk test. To compare independent samples, Student’s *t*-test was used for parametric data and Mann–Whitney U test for nonparametric data. To compare paired samples, Student’s t-test was used for parametric data and Wilcoxon test for non-parametric data. Fold-induction normalized data were evaluated using Student’s *t*-test for parametric samples and the Wilcoxon test for non-parametric samples, assigning the value of 1 to the control condition in each case. The differences in the clinical features between inactive and active patients were analyzed using the chi-square test or Fisher’s exact test. Statistical significance was defined as a *p*-value ≤ 0.05.

## 5. Conclusions

The present study contributes an improved understanding of the dysregulation of PSGL-1/P-selectin pathway in SLE patients leading to increased extracellular DNA accumulation in tissues. Our data suggest that PSGL-1/P-selectin axis could be a molecular target for designing more specific and effective treatments for SLE and thus avoiding general side effects.

## Figures and Tables

**Table 1:** Clinical features of SLE patients participating in the study performed to analyze the expression of PSGL-1 in neutrophils.

SLE Patients	Inactive SLE (*n* = 33)	Active SLE (*n* = 14)	*p*-Value
Female/Male, *n* (%)	30 (90)/3 (10)	13 (92)/1 (8)	
Age (years), mean [min–max]	50.35 [23–83]	43.28 [19–59]	
Disease duration (years), mean [min–max]	13.79 [1 month–38 years]	8.57 [1 month–21 years]	
ANA + profile, *n* (%)	32 (96)	14 (100)	
Anti-ds-DNA + profile, *n* (%)	13 (39)	13 (92) ***	0.0010
Anti-Sm + profile, *n* (%)	5 (15)	3 (21)	0.6795
Lupus anticoagulant	5 (15)	5 (35)	0.1373
SLICC/ACR Damage Index (mean [min–max])	0.57 [0–4]	0.71 [0–3]	0.1436
SLEDAI (mean [min–max])	1.11 [0–4]	14.14 [6–41] ****	<0.0001
Renal manifestations, *n* (%)	15 (45)	12 (85) *	0.0220
Lung manifestations, *n* (%)	5 (15)	5 (35)	0.1171
Raynaud´s phenomenon	6 (18)	3 (21)	0.5435
Treatment, *n* (%)			
Azathioprine	15 (45)	8 (57)	
Belimumab	2 (6)	4 (28)	
Glucocorticoids	29 (87)	13 (92)	
Hydroxychloroquine	30 (90)	14 (100)	
Methotrexate	9 (27)	4 (28)	
Mycophenolate mofetil	4 (11)	12 (85)	
Rituximab	6 (18)	5 (35)	

Abbreviations: SLE: systemic lupus erythematosus; ANA: antinuclear antibodies; Anti-ds-DNA: an-ti-double stranded DNA antibodies; Anti-Sm: anti Smith antibodies. SLICC/ACR: Systemic lupus international collaborating clinics/American College of Rheumatology; SLEDAI: SLE disease activity index. * *p* ≤ 0.05; *** *p* < 0.001; **** *p* < 0.0001.

**Table 2:** Clinical features and treatment of SLE patients participating in the in vitro experimental studies.

SLE Patients	Inactive SLE (*n* = 7)	Active SLE (*n* = 8)	*p*-Value
Female/Male, *n* (%)	7 (100)	7 (87)/1 (13)	
Age (years), mean [min–max]	48.85 [24–63]	29.87 [23–54]	
Disease duration (years), mean [min–max]	25 [5–38 years]	5.08 [1 month–16 years]	
ANA + profile, *n* (%)	6 (85)	8 (100)	
Anti-ds-DNA + profile, *n* (%)	2 (28)	8 (100) ***	0.0070
Anti-Sm + profile, *n* (%)	1 (14)	5 (62)	0.1189
Lupus anticoagulant	1 (14)	2 (25)	0.5515
SLICC/ACR Damage Index (mean [min–max])	0.57 [0–3]	1 [0–3]	0.1883
SLEDAI (mean [min–max])	2.85 (0–4)	14 (11–23) ***	0.0013
Renal manifestations, *n* (%)	4 (57)	7 (87)	0.2821
Lung manifestations, *n* (%)	1 (14)	3 (37)	0.3392
Raynaud´s phenomenon	1 (14)	2 (25)	0.5545
Treatment, *n* (%)			
Azathioprine	0 (0)	1 (12)	
Glucocorticoids	3 (42)	7 (87)	
Cyclophosphamide	0 (0)	1 (12)	
Hydroxychloroquine	2 (28)	6 (75)	
Methotrexate	1 (14)	2 (25)	
Mycophenolate mofetil	3 (42)	1 (12)	

Abbreviations: SLE: systemic lupus erythematosus; ANA: antinuclear antibodies; Anti-ds-DNA: an-ti-double stranded DNA antibodies; Anti-Sm: anti Smith antibodies. SLICC/ACR: Systemic lupus in-ternational collaborating clinics/American College of Rheumatology; SLEDAI: SLE disease activity index. *** *p* < 0.001.

## Data Availability

The original contributions presented in the study are included in the article/supplementary material, and further inquiries can be directed to the corresponding author.

## Supplementary Materials

The following supporting information can be downloaded at: https://www.mdpi.com/article/10.3390/ijms24076144/s1.
