# Supplementary material for: Low P-Selectin Glycoprotein Ligand-1 Expression in Neutrophils Associates with Disease Activity and Deregulated NET Formation in Systemic Lupus Erythematosus
Source: Int J Mol Sci. 2023 Mar 24;24(7):6144. doi: 10.3390/ijms24076144 (PMC10093849; doi:10.3390/ijms24076144)
Supplement: Supplementary file 1 [file ijms-24-06144-s001.zip › ijms-2201711-supplementary.pdf]

# Low P-Selectin Glycoprotein Ligand-1 expression in neutrophils associates with disease activity and deregulated NET formation in Systemic Lupus Erythematosus

**Supplementary Figures**

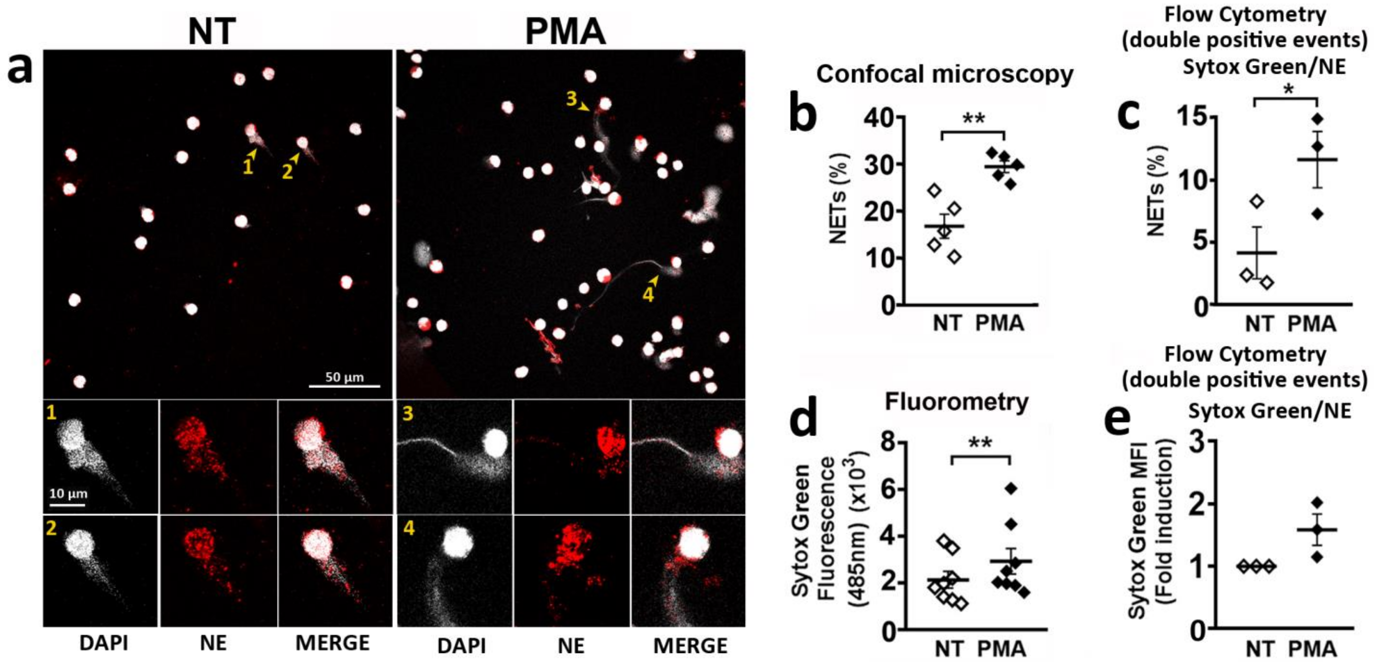


**Figure S1. Quantification of NETs induced by PMA**. Circulating neutrophils isolated from healthy donors were incubated with or without PMA (25 ng/mL) at 37ºC for 1 hour, on wells coated with 0.5% BSA. After incubation, samples were stained with Sytox Green, Sytox Green plus DAPI, Sytox Green plus NE or DAPI plus NE. **a**) Representative fields of DAPI/NE stained neutrophils obtained by confocal microscopy with a 63x objective. Scale bar=10-50µm. Yellow arrowheads indicate representative NETs. Yellow numbers correspond to NETosing cells amplified below the figure. **b**) NETs percentage stained with DAPI/NE quantified by confocal microscopy. **c**) Percentage of Sytox Green/NE double positive events. **d and e**) Extracellular DNA was analyzed: **d**) by fluorometry, as Sytox Green fluorescence intensity at 485 nm and **e**) by flow cytometry as Sytox Green mean fluorescence intensity (MFI) fold induction in Sytox Green/NE double positive events. Statistical analysis was performed using unpaired Student's t-test. ** p<0.01. Data are expressed as mean ± SEM or percentage ± SEM (n=8). NET: neutrophil extracellular trap. NT: Not treated. NE: Neutrophil elastase.

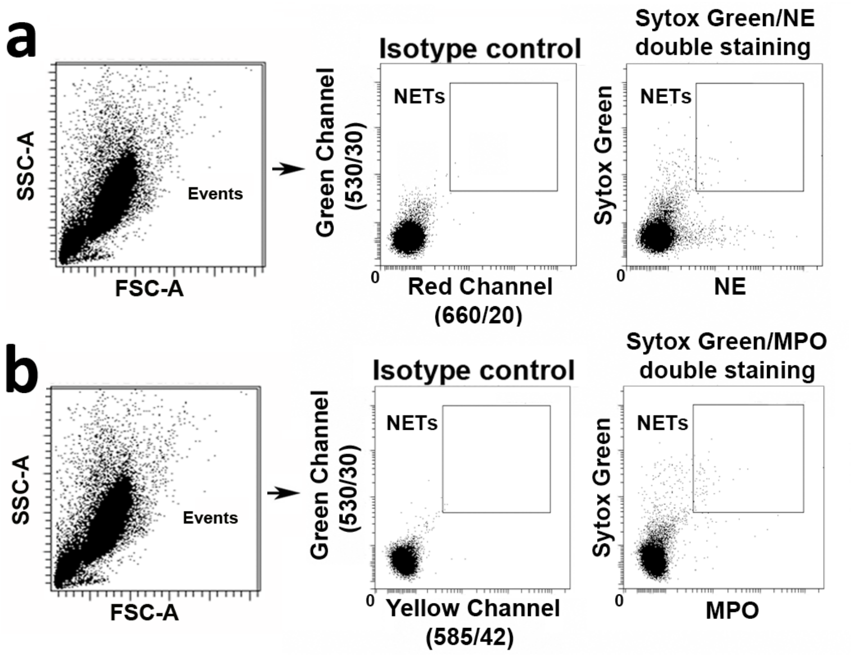


**Figure S2**. **Gating strategies for NETs identification by flow cytometry.** All events identified by SSC-A/FSC-A criteria, were considered for the analysis of NETs, which were then identified as: **a**) Sytox Green/NE-APC double positive events, by using an IgG-APC isotype control, or **b**) Sytox Green/MPO-PE double positive events, by using an IgG-PE isotype control. NE: Neutrophil elastase. MPO: Myeloperoxidase.

## Supplementary Tables

**Table S1.** Antibodies and probes for flow cytometry and confocal microscopy assays.

**Flow Cytometry**

| Antibody | Fluorochrome | Dilution | Company | Reference |
|---|---|---|---|---|
| anti-CD16 | APC-H7 | 1:100 | BD Pharmingen | 560195 |
| anti-CD162 (PSGL-1) | PE | 1:100 | BD Pharmigen | 556055 |
| Isotype-APC | APC | 1:100 | BD Pharmigen | 345818 |
| Isotype-APC-H7 | APC-H7 | 1:100 | BD Pharmigen | 561427 |
| Isotype-PE | PE | 1:100 | BD Pharmigen | 556656 |
| Isotype-FITC | FITC | 1:100 | BD Pharmigen | 551954 |
| Sytox Green* | Sytox Green | 1:25 | Invitrogen | S7020 |
| Phospho-Syk | PE | 1:50 | Cell Signaling | C87C1 |
| Neutrophil Elastase (NE) | APC | 1:100 | Novus Biologicals | 64392 |
| Myeloperoxidase (MPO) | PE | 1:100 | Miltenyi Biotec | 130-120-062 |
| anti-human CD62L (L-sel) | FITC | 1:100 | Biolegend | 304804 |
| **Confocal Microscopy** | | | | |
| anti-human CD162 (PSGL-1) | - | 1:100 | Biolegend | 328802 |
| Anti-Neutrophil Elastase | - | 1:200 | Abcam | ab68672 |
| Donkey anti-Rabbit IgG | Alexa Fluor 647 | 1:100 | Invitrogen | A31573 |
| Donkey anti-Mouse IgG | Alexa Fluor 555 | 1:100 | Invitrogen | A31570 |
| DAPI | DAPI | 1:20 | Sigma-Aldrich | D9542 |

* Same concentration was used for fluorometry.
